# Older menarche age and short reproductive period linked to chronic kidney disease risk

**DOI:** 10.1097/MD.0000000000015511

**Published:** 2019-05-03

**Authors:** Ji Hyun Noh, Hoseok Koo

**Affiliations:** a Department of Obstetrics and Gynecology; b Department of Medicine, Seoul Paik Hospital, College of Medicine, Inje University of Korea, Seoul, Republic of Korea.

**Keywords:** age, chronic kidney disease, menarche, reproductive period

## Abstract

This study aimed to investigate the association between reproductive period and menarche age and chronic kidney disease (CKD) in South Korean postmenopausal women.

This was a cross-sectional study of the data for 8510 postmenopausal women using the results of Korean National Health and Nutrition Examination Surveys over the past 6 years.

Of the total 8510 postmenopausal women, 790 (10.23%) were CKD patients. The menarche age in the CKD group was 16.2 ± 1.9 years old, which was higher than that in the non-CKD group (*P* < .001). The reproductive period of the CKD group was 32.4 ± 5.7 years, which was shorter than 33.3 ± 5.4 years in the non-CKD group (*P* < .001). The prevalence of CKD was 4.7% at a menarche age of 11 years or younger, which increased with increasing of menarche age, reaching 9.9% at menarche age of 16 years or older. According to the length of the reproductive period, the prevalence of CKD was 13.9% for the group less than 20 years of period and decreased significantly with increasing length of reproductive period. The prevalence of proteinuria was 7.2% in women with reproductive period of less than 20 years and significantly less in women with a reproductive period longer than 45 years (2.3%). The prevalence of CKD and proteinuria increased with increasing of menarche age, and the prevalence of CKD and proteinuria decreased with increasing of reproductive period.

The results suggest that CKD was associated with older menarche age and a short reproductive period. Management of life patterns and medical problems in women with old age at menarche and a short reproductive period should be considered.

## 1 Introduction

Chronic kidney disease (CKD) is a progressive and lifelong condition associated with various medical complications and affects about 5.9% of the Korean population worldwide.^[1]^ The prevalence of CKD is higher in older patients with diabetes, diabetes with hypertension, higher body mass index (BMI), higher systolic and diastolic blood pressures (BP), and higher hemoglobin A1c levels. Patients with CKD are more likely to be hospitalized or experience cardiovascular events such as cardiovascular mortality and noncardiac mortality, or all-cause mortality.^[2,3]^ The cost of medical care for CKD patients is 1.8 times that of patients without CKD. In 2010, the total Medicare used for end-stage renal disease in the United States exceeded 28 billion US dollars.^[4]^ Despite the high health and medical costs associated with CKD, the actual rate of awareness of kidney abnormalities is only 2.0% of the adult population.^[5]^

The causes of CKD are diabetes, hypertension, and glomerulonephritis, and the prognosis of CKD is affected by the degree of proteinuria control.^[6,7]^ Microalbuminuria, one of the diagnostic criteria for CKD, is a marker of renal vascular injury and a predictor of CVD.^[8]^ Several studies have examined the effects of estrogen on the presence or absence of progestin in microalbuminuria in women. One study showed that estrogen and progestin have no effect on microalbuminuria in postmenopausal women with type 2 diabetes mellitus (DM).^[9]^ In contrast, microalbuminuria was increased in pre-menopausal women using oral contraceptives and post-menopausal women receiving hormone replacement therapy compared with women who did not receive either of these medications.^[10,11]^

The level of estradiol (E2) levels is low in between 4 and 8 years and increased until 18 years. To the 46 years, the levels of E2 show plateau and decrease to the 54 years.^[12]^ Menarche is the first menstrual period experienced by a woman, and menarche age is influenced by several factors such as heredity, ethnicity, geography, and socio-economic environment, especially nutritional status.^[13–15]^ It is also related to health during the adult life.^[16–20]^ In recent decades, menarche age has steadily decreased among women, including Korean women.^[21]^ There were several studies about the association between menarche age and metabolic risk factors.^[18–20,22]^ Early (≤ age 10) and late menarche (≥ 16) represent a group at high risk for adult cardiometabolic abnormalities.^[18]^ A 1 year decrease in menarche age was associated with 0.81 (95% CI: 0.53, 1.08) greater risk of metabolic syndrome (*P* < .05 for all).^[19]^ A probable explanation for these associations is that menarche is followed by an increase in fat, and early menarche is associated with increased obesity and insulin resistance in adolescence, which may persist into adulthood.^[19,23]^

Women of reproductive age between 45 and 49 years old have a lower risk of CVD than men of similar age and lifestyle patterns,^[24]^ while those who experience early menopause have an increased risk of CVD.^[25]^ The mechanism of this association is unclear, but estrogen is thought to have a cardiovascular protective effect.^[26]^ Since the incidence of postmenopausal CVD increases significantly, fertile women can experience the cardiovascular protective effects of estrogens.^[24,27]^

Early menarche age increases the occurrence of obesity, type 2 DM, and metabolic syndrome, while the risk of CVD decreases. Further, a longer reproductive period is associated with more cardiovascular protection. Because CKD is associated with these factors, this study was designed with the goal of understanding the relationship between the effect of estrogen and the prevalence of CKD through menarche age and reproductive period.

## 2 Materials and methods

### 2.1 Study design and settings

In this cross-sectional, retrospective study, identified Korean female subjects were divided into 2 groups: non-CKD and CKD groups. For these groups, data of the Korea National Health and Nutrition Survey (KNHANES) for 6 years from 2010 to 2015 were used. The detailed design of the KNHANES is described in a previously published study.^[28]^

The survey was conducted by the Korean Ministry of Health and Welfare. It was composed of 3 sections including a health survey, health consultation, and a nutrition survey. The health survey was used to examine menarche age, menopause age, living area, educational status, household income, basic living status, marital status, pregnancy experience, pregnancy frequency, birth experience, first birth age, last childbirth age, breastfeeding experience, contraceptive use, alcohol, smoking, metabolic syndrome, myocardial infarction (MI), angina, and stroke. Health consultations were used to measure BP, abdominal circumference, BMI, hemoglobin, total cholesterol, triglyceride, high-density lipoprotein, low-density lipoprotein, serum creatinine, glomerular filtration rate, glucose levels, and proteinuria.

The non-CKD and CKD groups were composed of 7720 and 790 women, respectively, with a total of 8510 women. Of the 48,482 women identified for this study, 34,742 were excluded, of which 13,740 had no glomerular filtration rate and 26,232 had no data on menarche and menopause age. The reproductive period is defined as the difference between the menopause age and menarche age. Because these reproductive events occur within a narrow range of women's lives, reproductive period variables were grouped into 7 groups of 5-year increments.

### 2.2 Definitions

CKD was defined as an estimated GFR (eGFR) of < 60 mL/(min-1.73 m^2^), according to the definition in the Modification of Diet in Renal Disease (MDRD) study and as a person with proteinuria. eGFR was calculated using the 4-variable MDRD formula as follows: eGFR (mL/min·1.73 m^2^)=175 × serum creatinine level (mg/dL) 1.154 × age 0.203 × 0.742 (in women).^[29]^ DM was defined when a fasting plasma glucose level of ≥126 mg/dL was present or antidiabetic medication was administered. The definition of metabolic syndrome was based on the diagnosis category^[30]^ for the presence of ≥3 of the following: abdominal obesity of >90 cm in men or >80 cm in women; triglyceride level of ≥150 mg/dL or taking medication for triglyceride level; high-density lipoprotein cholesterol level of <40 mg/dL in men or <50 mg/dL in women, or taking medication; SBP ≥130 mm Hg and/or DBP ≥85 mm Hg or taking medication; fasting plasma glucose level of ≥100 mg/dL or taking medication. Thresholds for abdominal obesity were defined based on the Asian population.^[31]^ The prevalence of MI, angina, and stroke relied on answers from respondents to the health survey.

KNHANES was approved by the institutional review board of the Korea Centers for Disease Control (KCDC) and Prevention, and all participants provided written informed consent. The study protocol conformed to the ethics guidelines of the 1975 Declaration of Helsinki and was approved by the institutional review board of the KCDC (for 2010 to 2012, No. 2010–02CON- 21-C, 2011-02CON-06-C, 2012-01EXP- 01-2C, for 2013 to 2015, No 2013-07CON- 03-4C, 2013-12EXP- 03-5C, 2015-01-02-6C).

### 2.3 Statistical analyses

For continuous variables, descriptive analyses were performed to determine the subjects’ characteristics. Categorical variables were analyzed using the *χ*^2^ test to determine the correlation between different variables. The subjects were divided into 2 groups according to presence of CKD (CKD group versus non-CKD group). Differences in multiple groups (menarche age groups and reproductive period groups) means for the characteristics were determined using analysis of variance. In the regression analysis for the presence of CKD, menarche age, length of reproductive period, and confounding factors were adjusted, including BP, menarche age, menopause age, reproductive period, living area, educational status, household income, basic living status, marital status, pregnancy experience, pregnancy frequency, birth experience, first birth age, last childbirth age, breastfeeding experience, contraceptive use, abdominal circumference, BMI, hemoglobin, total cholesterol, triglyceride, high-density lipoprotein, low-density lipoprotein, serum creatinine, 1-man household, home ownership status, education level, drinking, smoking, metabolic syndrome, MI, angina, and stroke. Statistical significance was defined as a *P* < .001. R version 3.3.1. (R Foundation for Statistical Computing, Vienna, Austria) was used for the statistical analysis.

## 3 Results

### 3.1 Baseline characteristics

Of the 8510 participants, chronic renal disease was observed in 790 participants (10.23%). The results demonstrating differences in basic variables between the CKD group and non-CKD group are shown in Table 1. The menarche age of the CKD group was 16.2 ± 1.9 years old, and that of the non-CKD group was 15.6 ± 2.0 years old (*P* < .001). The menopause age in the CKD group was 48.4 ± 5.4 years old compared with the 49.0 ± 5.1 years old in the non-CKD group (*P* = .007). The reproductive period of the non-CKD group was 33.3 ± 5.4 years old, and that of the CKD group was the 32.4 ± 5.7 years old (*P* < .001). The CKD group had lower rates of living in the city, lower levels of education, lower incomes, and higher basic rates of receiving supply and demands. The non-CKD group had high level of education, more income, and more marriage. There was no significant difference in marriage, pregnancy, and childbirth, and breastfeeding experience and contraceptive use were higher in the CKD group. Abdominal circumference, BMI, and smoking rates in the CKD group were also higher.

**Table 1:**
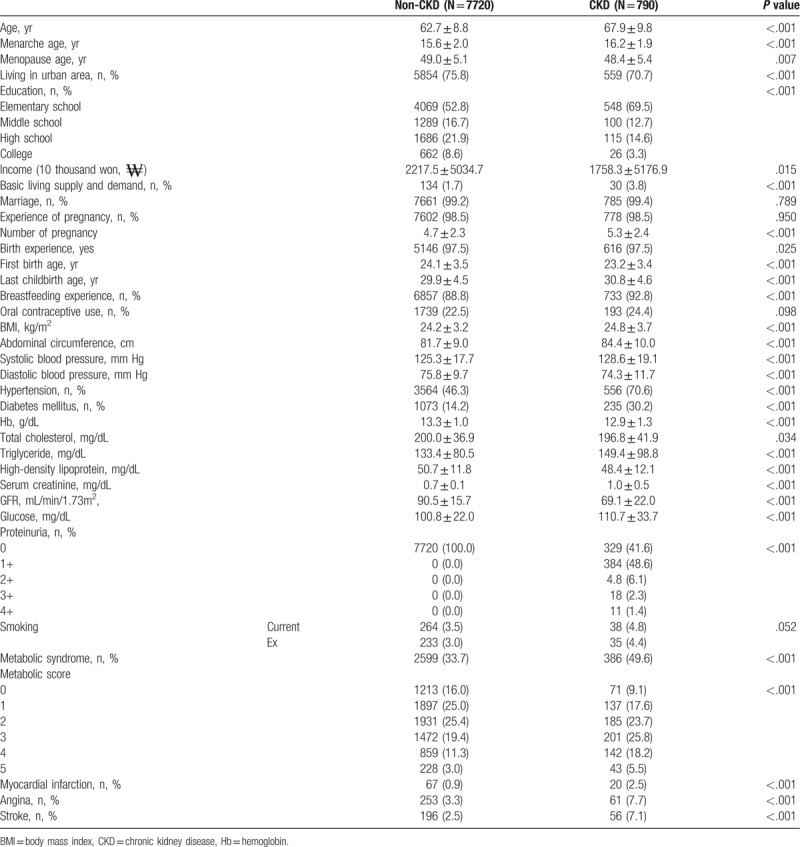
Baseline characteristics of patients according to CKD.

Furthermore, the CKD group had a higher rate of hypertension, diabetes, metabolic syndrome, MI, angina, and stroke than the non-CKD group (*P* < .001). Proteinuria was more common in CKD, and the CKD group had a GFR of 69.1 ± 22.0 mL/min/1.73 m^2^ (Table 1).

### 3.2 Relationship between menarche age and reproductive period according to CKD stage and proteinuria

Table 2 shows the relationship between menarche age, length of the reproductive period, CKD stage, and proteinuria. The prevalence of CKD was 4.7% when the menarche age was less than 11 years old. The prevalence of CKD increased as menarche age increased, reaching a rate of 9.9% when the menarche age was 16 years or older. There was no difference in the proportion of CKD stage 3 or higher. The prevalence of proteinuria was 5.6% higher in women with menarche after 16 years old compared with 4.7% of women with menarche below 11 years old. Therefore, the percentage of high proteinuria also increased in women with menarche after 16 years old. The prevalence rate of CKD was 13.9% for reproductive periods less than 20 years and 2.3% for reproductive periods longer than 45 years. The prevalence of CKD decreased markedly with a longer reproductive period. The prevalence of proteinuria was 7.2% in women whose reproductive period was less than 20 years and 2.3% in women with reproductive periods 45 years or longer. Therefore, the prevalence of proteinuria was significantly lower with longer reproductive period.

**Table 2:**
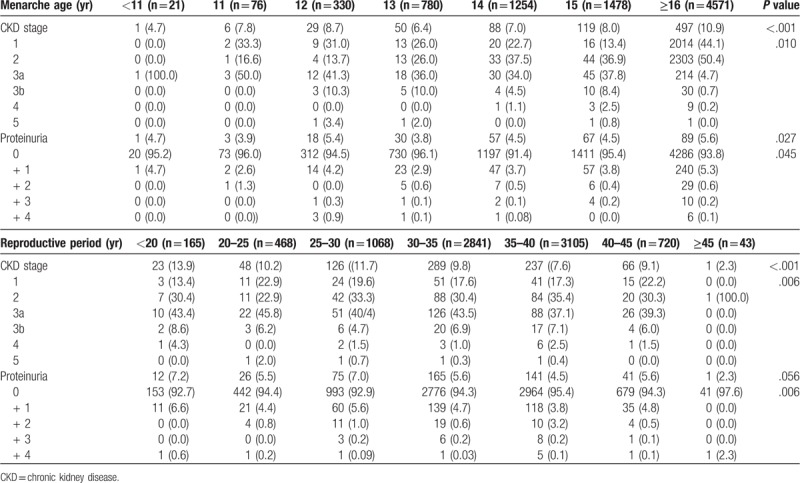
Relationship between menarche age and reproductive period according to CKD stage and proteinuria.

### 3.3 Relationship between vascular disease, menarche age, and reproductive period

Table 3 demonstrates the relationship between vascular disease and menarche age and length of reproductive period. The prevalence rate of metabolic syndrome and stroke was lower among women with menarche at 11 to 13 years old, but increased for women with menarche at age 14 to 16 or higher. The prevalence of angina and MI was not significantly different for the various ages at menarche, but an increased rate was seen among women with menarche at age 16 or higher. The relationship between metabolic syndrome and stroke was parabolic (U-shaped) based on menarche age, while MI and angina were not different based on menarche age and increased at age 16 years. The prevalence of CKD was significantly lower in patients with menarche below age 11 and was higher in patients with menarche at age 16 or higher. Hypertension was 52.9% higher in women with menarche at age 16 or higher compared with 42.9% in women with menarche below age 11. Type 2 DM was 28.6% higher in women with menarche under age 11 compared with 16.69% in women with menarche at age 16 or higher.

**Table 3:**
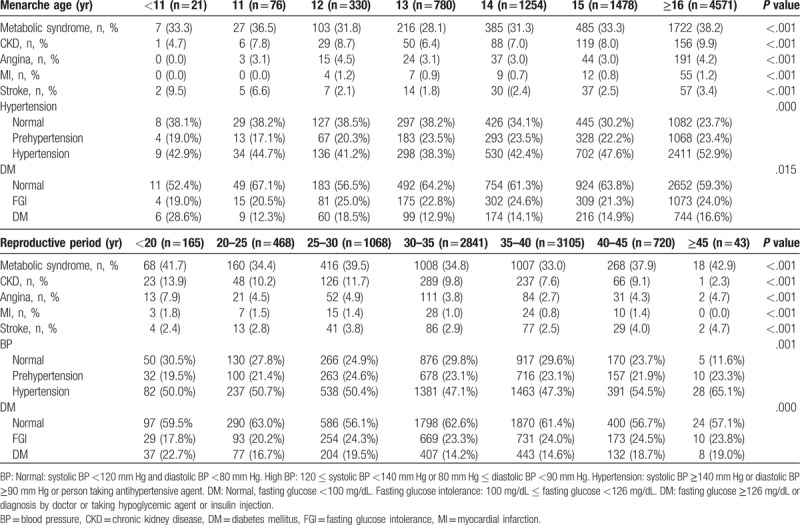
Relationship between vascular disease, menarche age, and reproductive period.

**Figure 1:**
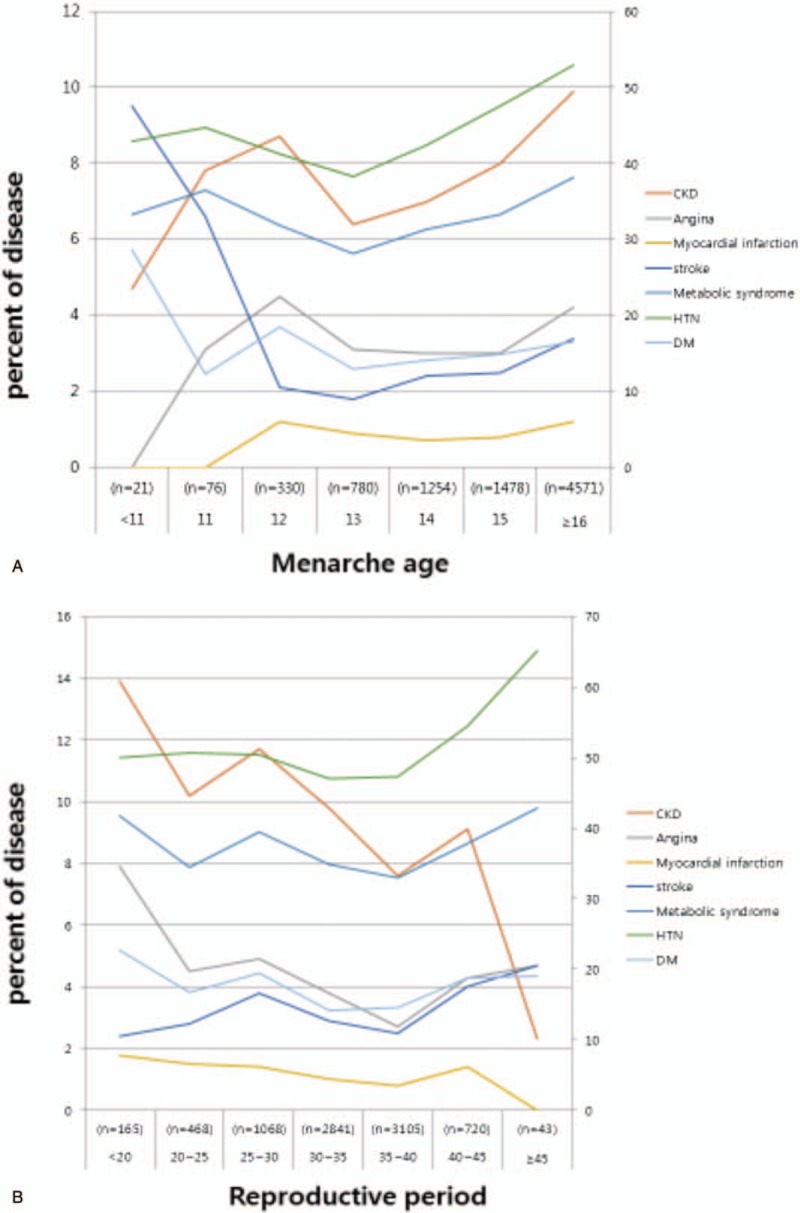
Prevalence of vascular disease according to menarche age and reproductive period. A, Prevalence of vascular disease according to menarche age. The prevalence of CKD was increased at the higher menarche age and decreased at the lower menarche age. B, Prevalence of vascular disease according to reproductive period. The prevalence of CKD was decreased at the longer reproductive period and increased at the shorter reproductive period. CKD = chronic kidney disease.

According to the reproductive period, the prevalence of metabolic syndrome was as high as 41.7% among women who had a reproductive period shorter than 20 years. The occurrence of metabolic syndrome fluctuated with increasing reproductive period, reaching a rate of 42.9% when the reproductive period was 45 years or longer. Furthermore, the prevalence rate of angina was 7.9% among those who had a reproductive period of less than 20 years and decreased as the length of the period increased. The rate of angina was the lowest at 3.8% among individuals whose reproductive period was between 35 and 40 years, but it slightly increased to 4.7% once the reproductive period was 45 years or longer. The prevalence of MI was 1.8% for individuals whose reproductive period was less than 20 years, and it decreased as the length of the period increased. MI was not reported among patients whose reproductive period was greater than or equal to 45 years. The prevalence of stroke increased from 2.4% for the group whose reproductive period was less than 20 years to 4.7% in the group whose reproductive period was greater than 45 years. The prevalence of CKD was 13.9% for individuals with the shortest reproductive period, and it decreased as the reproductive period increased, decreasing to 2.3% with a reproductive period of 45 years or longer. A 50% occurrence of hypertension was observed among patients in the group whose reproductive period was less than 20 years and this rate increased to 65.1% when the length of the reproductive period reached 45 years or longer. Finally, DM was 22.7% for the group whose reproductive period was shorter and 19% for the group who had a longer reproductive period. Figure 1 shows the prevalence of vascular diseases according to menarche age and reproductive period (Table 3). The prevalence of CKD and proteinuria increased with increasing menarche age, and the prevalence of CKD and proteinuria decreased with increasing length of reproductive period.

### 3.4 CKD risk according to menarche age and reproductive period

**Figure 2:**
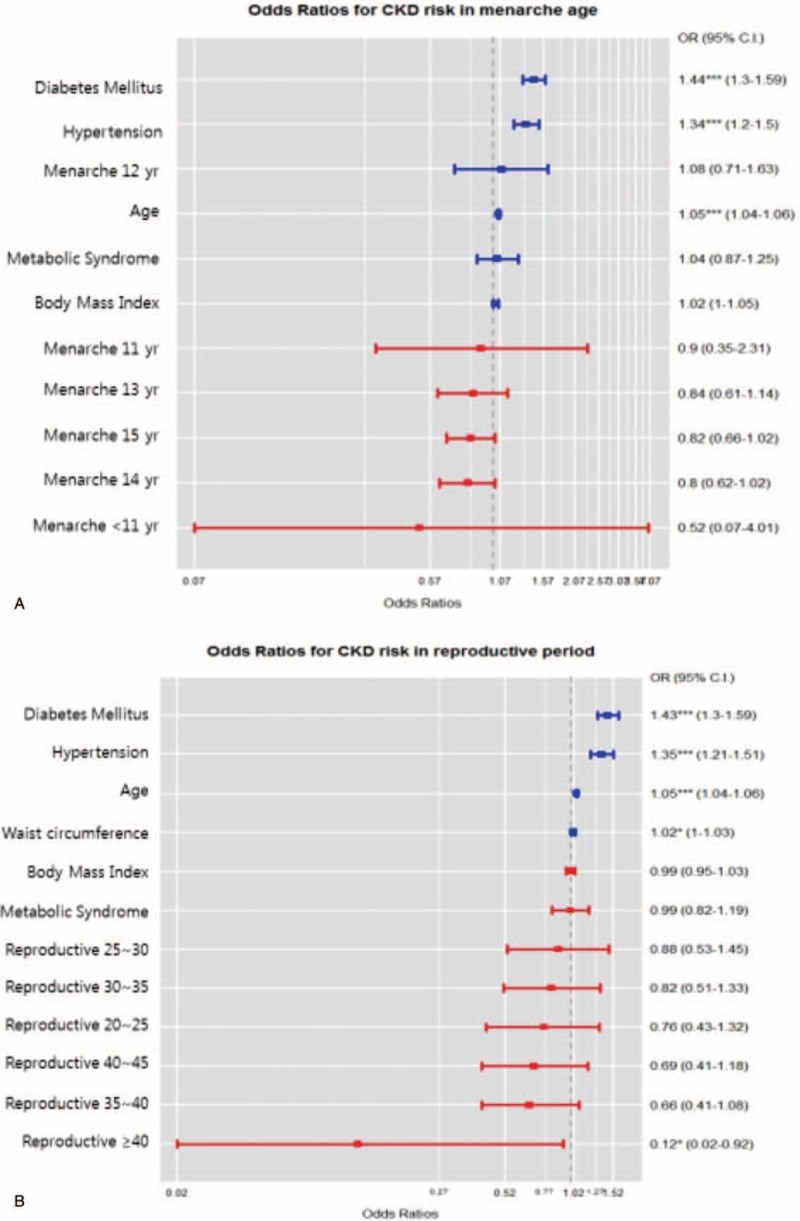
The odds ratios for risk of chronic kidney disease according to menarche age and reproductive period. A, Odds ratios according to menarche age. The risk of CKD was lower in women with menarche below age 11, but statistical significance was not observed. B, Odds ratios according to reproductive period. The risk of CKD was lower in women exhibiting a reproductive period of over 40 years, and statistical significance was observed. CKD = chronic kidney disease.

The risk of CKD according to menarche age was compared with other influencing variables. The risk of CKD was lower in women with menarche below age 11, but statistical significance was not observed (Fig. 2A OR; 0.52, 95% CI 0.07–4.01).

The risk of CKD according to reproductive period was compared with other influencing variables. The risk of CKD was lower in women exhibiting a reproductive period of over 40 years, and statistical significance was observed (Fig. 2B OR; 0.12, 95% CI 0.02–0.92).

## 4 Discussion

This study found that a longer reproductive period and early menarche are associated with association with respect to CKD in premenopausal Korean women. The prevalence rate of CKD was the highest at 13.9% when subjects had a reproductive period of less than 20 years. With increased reproductive period, the prevalence rate of CKD decreased. It was the lowest at 2.3%, and this was seen among women who had a reproductive period of over 45 years. The prevalence of CKD was decreased to 4.7% at menarche age below 11 years, and the higher the menarche age, the higher the prevalence of CKD and the stage of CKD. These results suggest that the longer that estrogen is released, the more protected the kidneys will be. Although the mechanism for explaining this association is unclear, it is believed that subsequent estrogenic actions will have kidney-protective effects. Estrogen can inhibit the progression of glomerulosclerosis by inhibiting collagen synthesis in glomerular mesangial cells.^[32–34]^ Gava et al^[33]^ reported that estrogen indirectly inhibits the proliferation of glomerular mesangial cells by regulating the synthesis of growth promoters and inhibitors. Animal models for CKD demonstrated slow renal injury progression in women and that proteinuria and glomerular fibrosis were reduced with estrogen therapy.^[35–38]^ According to the Berg study, the differences in renal progression between men and women accounted for the possible role of estrogen in a study consisting of 122 kidney donors.^[39]^ According to a cross-sectional and 10-year prospective study of postmenopausal estrogen therapy by Fung et al,^[40]^ estrogen users were reported to have better GFR and BP than nonusers. These results suggest that the longer the period of estrogen release, the longer the time for estrogen to protect the kidneys and a lower occurrence of CKD.

Microalbuminuria, one of the diagnostic criteria for CKD, is a marker of renal vascular injury and a predictor of CVD.^[8]^ Estrogen reduces low-density lipoprotein cholesterol, increases high-density lipoprotein cholesterol, has beneficial effects on lipid parameters, promotes nitric oxide-mediated vasodilation, inhibits blood vessel response to injury, and inhibits atherosclerosis progression.^[41]^ The prevalence rate of proteinuria was the highest at 7.2% when the reproductive period was shorter than 20 years, and this occurrence was lowered with longer reproductive period; it reached its lowest rate at 2.3% when the length of the period was over 45 years.

In this study, vascular disease was significantly lower in patients with CKD and MI when the reproductive period was longer than 45 years. The prevalence rate of metabolic disease and angina was the lowest when the reproductive period was between 35 and 40 years, and the prevalence rate of stroke was the highest in over 45 years (Fig. 1B, *P* < .001). The prevalence rate of angina, metabolic syndrome, and stroke was low when the reproductive period was between 35 and 40 years. CKD, MI, metabolic syndrome, and angina showed the highest prevalence rate in the group whose reproductive period was less than 20 years. The prevalence of these vascular diseases was lowered with longer reproductive period, and this may be related to the vascular protective effect of estrogen on arterial vasodilation by activating endothelial cell nitric oxide synthase.

Currently, the prevalence rate of CKD in Korea ranges from 8.9% to 13.7%, and the percentage of women experiencing CKD is 11.0%.^[42–44]^ No other studies have compared the prevalence of CKD before and after menopause. In this study, the prevalence of CKD was 4.7% when the menarche age was below 11 years old. The prevalence rate increased with increasing menarche age, reaching 9.9% when the menarche age was 16 years or older; in other words, the longer the reproductive period, the lower the prevalence rate of CKD. To explain the effect of estrogen on the vascular protection effect of proteinuria, proteinuria was increased as the menarche age increased, and proteinuria decreased as the length of the reproductive period increased. Important factors in the cause and progression of CKD are DM, hypertension, age, metabolic syndrome, and obesity.^[45]^ In this study, when comparing the menarche age and length of the reproductive period with the factors affecting the development of CKD, there was no difference in age-related risk for menarche age. However, when the length of the reproductive period was compared with other factors, a lower risk of CKD was observed. A probable reason for this observation is that a more complicated mechanism is involved in menarche age. The association between early menarche and increased CVD risk factors such as abdominal obesity, hypertension, diabetes, and metabolic syndrome can be explained by several different mechanisms. Menarche is followed by an increase in fat, and early menarche is associated with pubertal obesity and insulin resistance, which persist until adulthood.^[19,23]^

The effects of estrogen on hypertension and diabetes are known. With lower menarche age, the effect of estrogen is prolonged, more mast cells grow, the risk of obesity and diabetes is increased, and prevalence of hypertension and prevalence and mortality of CVD are increased.^[19,46,47]^ In this study, the prevalence of hypertension, MI, angina, and CKD was found to increase with older menarche age. DM was more prevalent with younger menarche age. On the contrary, the relationship between the length of the reproductive period and disease is not well known. In this study, MI, angina, and CKD were less prevalent with a longer reproductive period. However, the prevalence of hypertension was different to what prior studies indicate, and it was thought that age could have an effect on the prevalence of hypertension.

This research has some limitations. First, the cross-sectional study design limits the ability to determine causal relationships. Causes other than CKD risk and role of estrogen are possible. Second, given the retrospective design, the women may have errors in recalling their menarche and menopause age. Third, there is a difference between menarche age and reproductive period and the occurrence of hypertension. Age was not adjusted for hypertension, which is one of the risk factors for CKD. Fourth, the data of Menarche age, menopause age was dependent on the survey, the characteristics of KHANES study. So there can be a recall error. The strength of this study is the sample size, it is larger than the previous study. And we also observed the menarche age and reproductive period by subdivision.

Nonetheless, the study is meaningful in that it is the first study to investigate the prevalence of CKD according to menarche age and length of reproductive period in a large group of Korean women. In addition, more attention should be credited to the health care of women with late menarche and a short reproductive period. Furthermore, more studies should be conducted regarding diseases associated with menarche age and length of reproductive period.

In conclusion, higher CKD prevalence was associated with older age at menarche and a short reproductive period. Management of life patterns and medical problems in women with older age at menarche and a short reproductive period should be considered.

## Author contributions

**Data curation:** Ji Hyun Noh.

**Formal analysis:** hoseok koo, Ji Hyun Noh.

**Methodology:** hoseok koo, Ji Hyun Noh.

**Writing – original draft:** Ji Hyun Noh.

**Writing – review & editing:** hoseok koo.
